# PSME2 identifies immune-hot tumors in breast cancer and associates with well therapeutic response to immunotherapy

**DOI:** 10.3389/fgene.2022.1071270

**Published:** 2022-12-13

**Authors:** Cen Wu, Ren Zhong, Xiaofei Sun, Jiajie Shi

**Affiliations:** ^1^ Department of General Surgery, Rudong County People’s Hospital, Nantong, China; ^2^ Departments of Breast Oncology, The Fourth Hospital of Hebei Medical University, Shijiazhuang, China

**Keywords:** breast cancer, PSME2, biomarker, bioinformatics, time feature

## Abstract

Breast cancer (BrCa) is a heterogeneous disease, which leads to unsatisfactory prognosis in females worldwide. Previous studies have proved that tumor immune microenvironment (TIME) plays crucial roles in oncogenesis, progression, and therapeutic resistance in Breast cancer. However, biomarkers related to TIME features have not been fully discovered. Proteasome activator complex subunit 2 (PSME2) is a member of proteasome activator subunit gene family, which is critical to protein degradation mediated by the proteasome. In the current research, we comprehensively analyzed the expression and immuno-correlations of Proteasome activator complex subunit 2 in Breast cancer. Proteasome activator complex subunit 2 was significantly upregulated in tumor tissues but associated with well prognosis. In addition, Proteasome activator complex subunit 2 was overexpressed in HER2-positive Breast cancer but not related to other clinicopathological features. Interestingly, Proteasome activator complex subunit 2 was positively related to immune-related processes and identified immuno-hot TIME in Breast cancer. Specifically, Proteasome activator complex subunit 2 was positively correlated with immunomodulators, tumor-infiltrating immune cells (TIICs), immune checkpoints, and tumor mutation burden (TMB) levels. Moreover, the positive correlation between Proteasome activator complex subunit 2 and PD-L1 expression was confirmed in a tissue microarray (TMA) cohort. Furthermore, in an immunotherapy cohort of Breast cancer, patients with pathological complete response (pCR) expressed higher Proteasome activator complex subunit 2 compared with those with non-pathological complete response. In conclusion, Proteasome activator complex subunit 2 is upregulated in tumor tissues and correlated with the immuno-hot tumor immune microenvironment, which can be a novel biomarker for the recognition of tumor immune microenvironment features and immunotherapeutic response in Breast cancer.

## Introduction

Breast cancer (BrCa) is one of the most widespread cancerous diseases in females worldwide, which has been a significant threat to women’s health (Siegel et al., 2022). Being a heterogeneous tumor, BrCa is divided into four various molecular subtypes according to genetic profiles, including Basal-like, Luminal-A, Luminal-B, and human epidermal growth factor 2 (HER2)-positive BrCa (Yeo and Guan, 2017). The molecular subtype has a significant influence on therapeutic regimen and prognosis of BrCa. Up to now, the main therapeutic strategy of BrCa involves surgical resection, chemotherapy and radiotherapy, endocrine therapy, immunotherapy, or the combination of them (Greenlee et al., 2017). However, amass of patients with advanced BrCa still have poor prognosis, and lack effective therapies to prevent the progression.

Recently, increasing numbers of researchers have focused on cancer immunotherapy, which has exerted significant effects on the therapeutic situation of many malignancies (Vesely et al., 2022). Immunotherapy is defined as that drugs are used to activate the natural mechanisms of the immune system, to kill cancer cells (Zhang et al., 2022a). Encouragingly, immunotherapy has become one of the major therapeutic strategies in BrCa. Immune checkpoint inhibitors combined with chemotherapy have demonstrated significant efficacy in both early-stage and advanced triple-negative breast cancer (Abdou et al., 2022). Emerging data from immunotherapy studies in advanced hormone receptor-positive as well as HER2-positive BrCa are arising with mixed results (Cejuela et al., 2022).

However, in some cases, immunotherapy does not have satisfactory responses. Scholars have found that the tumor immune microenvironment (TIME) has a significant influence on the efficacy of immunotherapy (Frankel et al., 2017). TIME is composed of various cellular and non-cellular components, including tumor cells, immune cells, stromal cells, cytokines and vascular networks (Cai et al., 2021). TIME is heterogeneous, and different density and diversity of tumor-infiltrating immune cells (TIICs) are vital causes of diverse prognosis when patients receive immunotherapy (Bejarano et al., 2021). The TIME could be simply divided into cold or hot according to its characteristics (Duan et al., 2020). Hot tumors exhibit high level of immune cell infiltration in tumor tissues and immuno-activation, and cold tumors are characterized by notable features of immune cells absence or exclusion (Duan et al., 2020). Usually, hot tumors are sensitive to immunotherapy. Herein, biomarkers related to the TIME features are essential to predict and evaluate the immunotherapeutic response.

Proteasome activator complex subunit 2 (PSME2), a member of proteasome activator subunit gene family, is located on 14q12, and expresses mainly intracellular (Wang et al., 2021). The protein encoded by *PSME2* gene is critical to protein degradation mediated by the proteasome (Wang et al., 2004). Diseases associated with PSME2 abnormality include Immunodeficiency 12 and Ulceroglandular Tularemia (Guo et al., 2021). PSME2 is highly expressed in liver, pancreas and lung. Many studies have reported the prognostic value of proteasome activator subunit gene family in cancers (Qin et al., 2019a; Qi et al., 2021; Zheng et al., 2022). However, the expression and immune features of PSME2 in BrCa have not been revealed.

In this study, we used the BrCa cohort from the Cancer Genome Atlas (TCGA) dataset to investigate the expression and immunological characteristics of PSME2 in BrCa. We come to the conclusion that PSME2 is positively related to expression of immunological factors and immune-related processes. Moreover, an in-house cohort and an immunotherapy BrCa cohort were also used as further validation of immunological characteristics of PSME2. Overall, it was demonstrated that high PSME2 expression is tightly connected with an inflamed TIME, which could be used to identify BrCa patients who are sensitive to immunotherapy.

## Methods

### Public datasets acquisition

Standardized RNA-seq and clinical data of BrCa samples from the TCGA dataset were acquired from the UCSC Xena (https://xenabrowser.net/datapages/). In addition, the GSE173839 dataset (Pusztai et al., 2021), an immunotherapy cohort was acquired from the Gene Expression Omnibus (GEO, https://www.ncbi.nlm.nih.gov/geo/) data storage. For the immunotherapy cohort, diagnostic patients who received immunotherapy were selected for further analysis.

### Correlation genes screen and enrichment analysis

Linked Omics (https://www.linkedomics.org/login.php) is an interactive platform utilized to manage TCGA data online (Vasaikar et al., 2018). In the current study, the Linked Omics was utilized to screen genes that correlated with PSME2 expression in BrCa. The functional roles of PSME2 in BrCa were predicted using the Linked Omics platform in terms of Biological Process (BP) and Kyoto Encyclopedia of Genes and Genomes (KEGG) analysis by the gene set enrichment analysis (GSEA). Default options were utilized for all parameters.

### Analysis of the correlation between PSME2 and TIME features

The features of the TIME were mainly reflected based on the expression levels of immunomodulators, including chemokines, MHC, receptors, immunoinhibitors, and immunostimulators. In addition, TIICs levels estimated by the TIMER algorithm (Li et al., 2017), immune checkpoints expression levels and immunophenoscore (IPS) (Charoentong et al., 2017) were also considered as the features of TIME. According to a previous study, IPS was calculated using machine learning by consideration of the four major categories of componentsassociated with immunogenicity: effector immune cells, immunosuppressive immune cells, MHC molecules, and immunomodulators (Charoentong et al., 2017). The IPS values of BrCa patients were downloaded from the Cancer Immunome Atlas (TCIA) (https://tcia.at/home). Moreover, the simple nucleotide variation data of the TCGA samples processed by the “MuTect2” software (Beroukhim et al., 2010) was obtained from the Genomic Data Commons (GDC, https://portal.gdc.cancer.gov/) website. We used the R package “maftools” to visualize mutation landscape and calculate the tumor mutation burden (TMB) of each sample. In addition, the T cell inflamed score was calculated based on the linear combination of the expression levels and weighting coefficient of 18 genes (Ayers et al., 2017). In conclusion, correlations between PSME2 expression and immunomodulators, TIICs, IPS, TMB, and T cell inflamed score were evaluated. The most commonly used 50% as the cut-off value for PSME2 high or low expression in the current study.

### Clinical cohorts

The BrCa (Cat. HBreD050Bc01) tissue microarray (TMA) was obtained from Outdo BioTech (Shanghai, China). The HBreD050Bc01 cohort contained 40 tumor samples and 10 para-tumor samples. Specific clinic-pathological characteristics were obtained from Outdo BioTech. Ethical approval for the use of TMA was granted by the Clinical Research Ethics Committee in Outdo Biotech (Shanghai, China).

### Immunohistochemistry and semi-quantitative evaluation

Immunohistochemistry (IHC) staining was performed on the above TMAs. The primary antibodies used in the research were as follows: anti-PSME2 (1:1000 dilution, Cat. 12937-2-AP, ProteinTech, Wuhan, China) and anti-PD-L1 (Ready-to-use, Cat. GT2280, GeneTech, Shanghai, China). Antibody staining was visualized with DAB and hematoxylin counterstain. All stained sections were independently assessed by two pathologists. For semi-quantitative assessment of PSME2 and PD-L1 staining, the immunoreactivity score (IRS) was used (Cai et al., 2021). Briefly, the percentage of positively stained cells was scored as 0–4: 0 (<5%), 1 (6–25%), 2 (26%–50%), 3 (51%–75%) and 4 (>75%). The staining intensity was scored as 0–3: 0 (negative), 1 (weak), 2 (moderate), and 3 (strong). The IRS equals the percentages of positive cells multiplied by staining intensity.

### Statistical analysis

Statistical analysis and figure production were performed using R language 4.0.2, Graphpad Prism 6.0 and SangerBox (Shen et al., 2022). R packages, including maftools, pheatmap, corrplot, psych, and ggpubr were used for statistical analysis and figure production. The statistical difference of continuous variables between the two groups was assessed by the Student t test or Mann-Whitney test. Prognostic values of categorical variables were evaluated by log-rank test. Pearson’s correlation was utilized to evaluate the correlation between two variables. Receiver-operating characteristic (ROC) analysis was utilized to appraise the specificity and sensitivity of PSME2 in predicting immunotherapeutic responses, and the area under the ROC curve (AUC) was calculated. The association between PSME2 expression and the immunotherapeutic responses was assessed using Pearson’s Chi-squared test. For all analyses, *p*-value <0.05 was deemed to be statistically significant.

## Results

### PSME2 was upregulated in tumor but predicted well prognosis in BrCa

**FIGURE 1:**
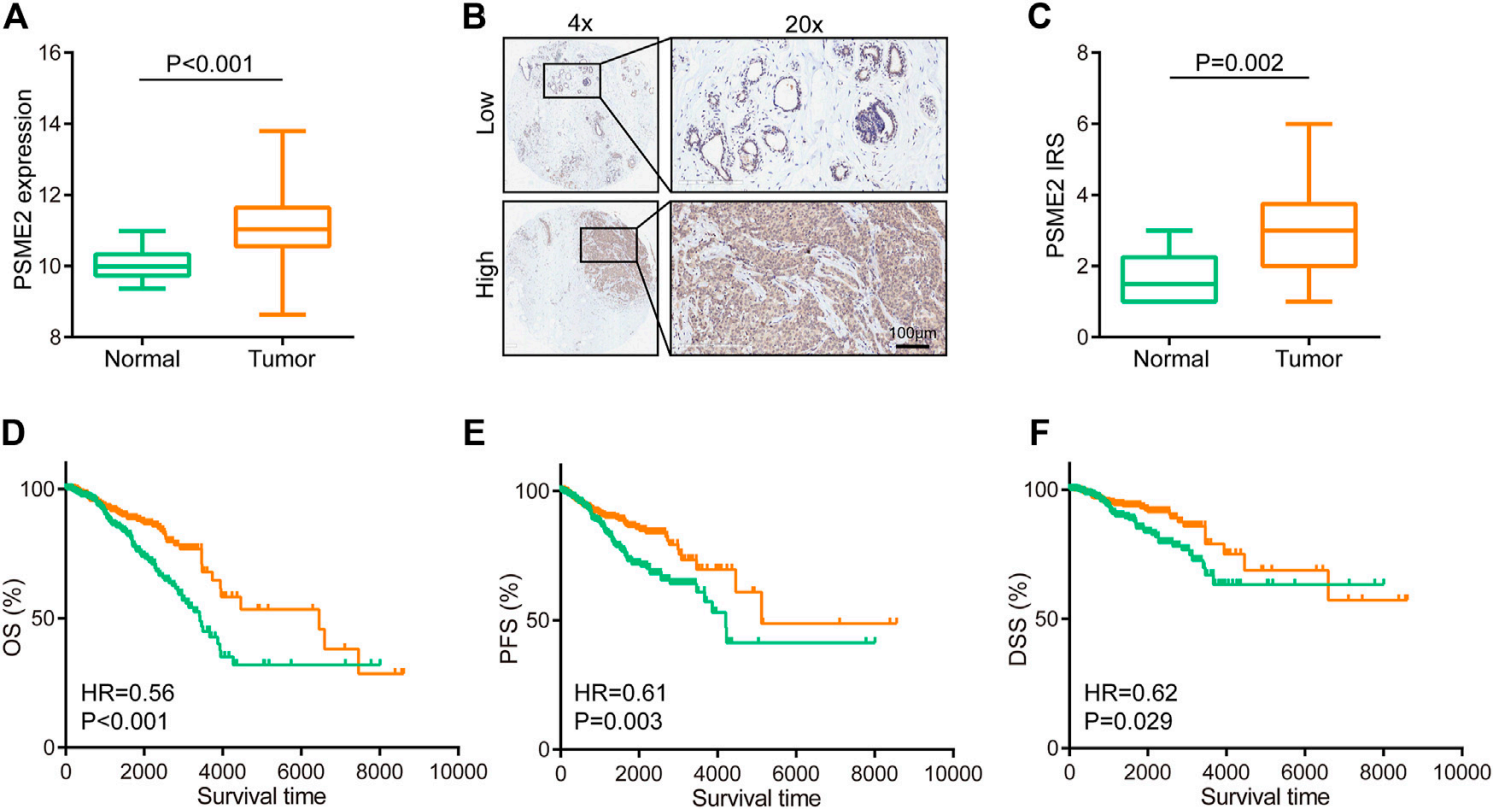
Expression and prognostic value of PSME2 in BrCa **(A)**Expression level of PSME2 in tumor and para-tumor tissues. Compared with para-tumor tissues, PSME2 was highly expressed in tumor tissues. Data was obtained from the TCGA dataset **(B)** Representative images revealing PSME2 expression in tumor and para-tumor tissues using anti-PSME2 staining **(C)** Expression level of PSME2 protein in tumor and para-tumor tissues. Compared with para-tumor tissues, PSME2 was highly expressed in tumor tissues **(D–F)** Prognostic value of PSEM2 in BrCa in terms of OS, PFS, and DFS. High expression of PSEM2 was associated with well prognosis in BrCa. Data was obtained from the TCGA dataset.

We used the BrCa cohort from the TCGA dataset to compare the mRNA levels of PSME2 between BrCa and para-tumor breast tissues. The analysis revealed that PSME2 is remarkably overexpressed in BrCa tissues (Figure 1A). To further validate this finding, we next conducted IHC staining in 40 BrCa and 10 normal samples to analyze PSME2 expression. Most cells of BrCa samples were stained with anti-PSME2 antibodies. On the contrary, the majority of the cells in para-tumor samples were not stained (Figure 1B). The result confirmed that expression of PSME2 in BrCa was higher than that in para-tumor tissues (Figure 1C). To assess the prognostic value of PSME2, the TCGA dataset was utilized to download survival data which included three endpoints, including overall survival (OS), progression-free survival (PFS), and disease-specific survival (DSS). The results of survival analysis showed that high mRNA expression of PSME2 was remarkably related to better OS, PFS, and DSS of BrCa patients. Overall, PSME2 expression is increased in BrCa compared with para-tumor tissues, but predicts well prognosis in BrCa.

### Associations between PSME2 and clinicpathological parameters of BrCa

**FIGURE 2:**
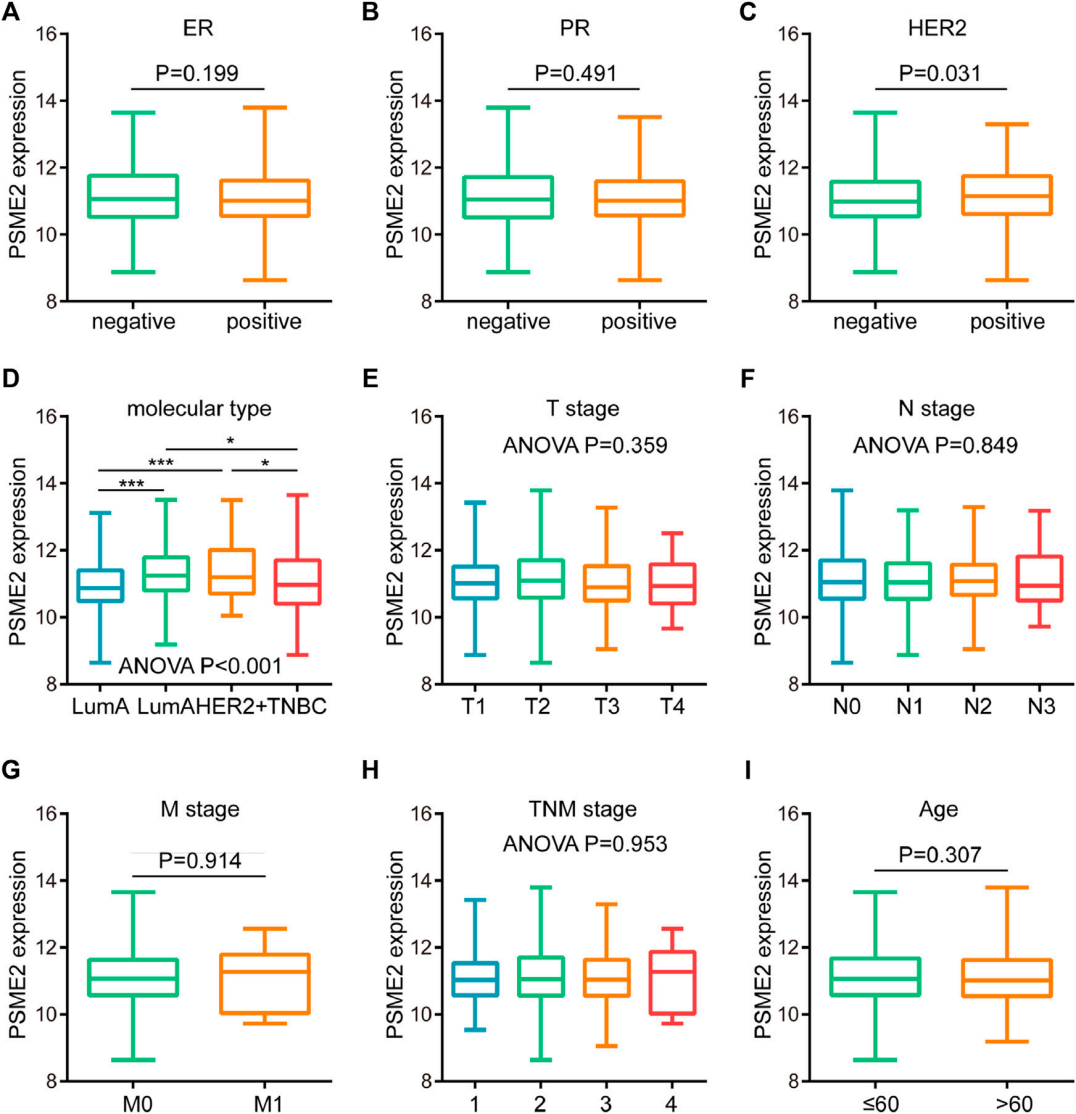
Associations between PSME2 and clinicopathological features in BrCa. Expression level of PSME2 in BrCa with different **(A)** ER **(B)** PR **(C)** HER2 status **(D)** molecular subtypes **(E)** T **(F)** N **(G)** M **(H)** clinical stages, and **(I)** ages. PSME2 expression was only associated with HER2 status and molecular types. Data was obtained from the TCGA dataset. *p-value <0.05, and ***p-value <0.001.

Since PMSE2 was highly expressed in BrCa, we speculated that overexpression of PSME2 may be related to several clinicopathological parameters of BrCa patients. Hence, we analyzed the associations between PSME2 and clinicpathological features of BrCa patients, including age, TMN stage and molecular type. As shown in Figure 2, PSME2 expression had no relationship with expression level of ER and PR status, but was clearly correlated with HER2 status (Figures 2A–C). The relationship between molecular type and PSME2 expression also confirms that PSME2 was overexpressed in HER2-ponsitive tumors (Figure 2D). In the meantime, PSME2 expression level had no relationship with TMN stage and age (Figures 2E–I). Thus, we come to the conclusion that PSME2 is promising to be used as a novel biomarker to predict the molecular type of BrCa, and has no correlation with other clinical features.

### PSME2 was positively related to immune-related processes in BrCa

**FIGURE 3:**
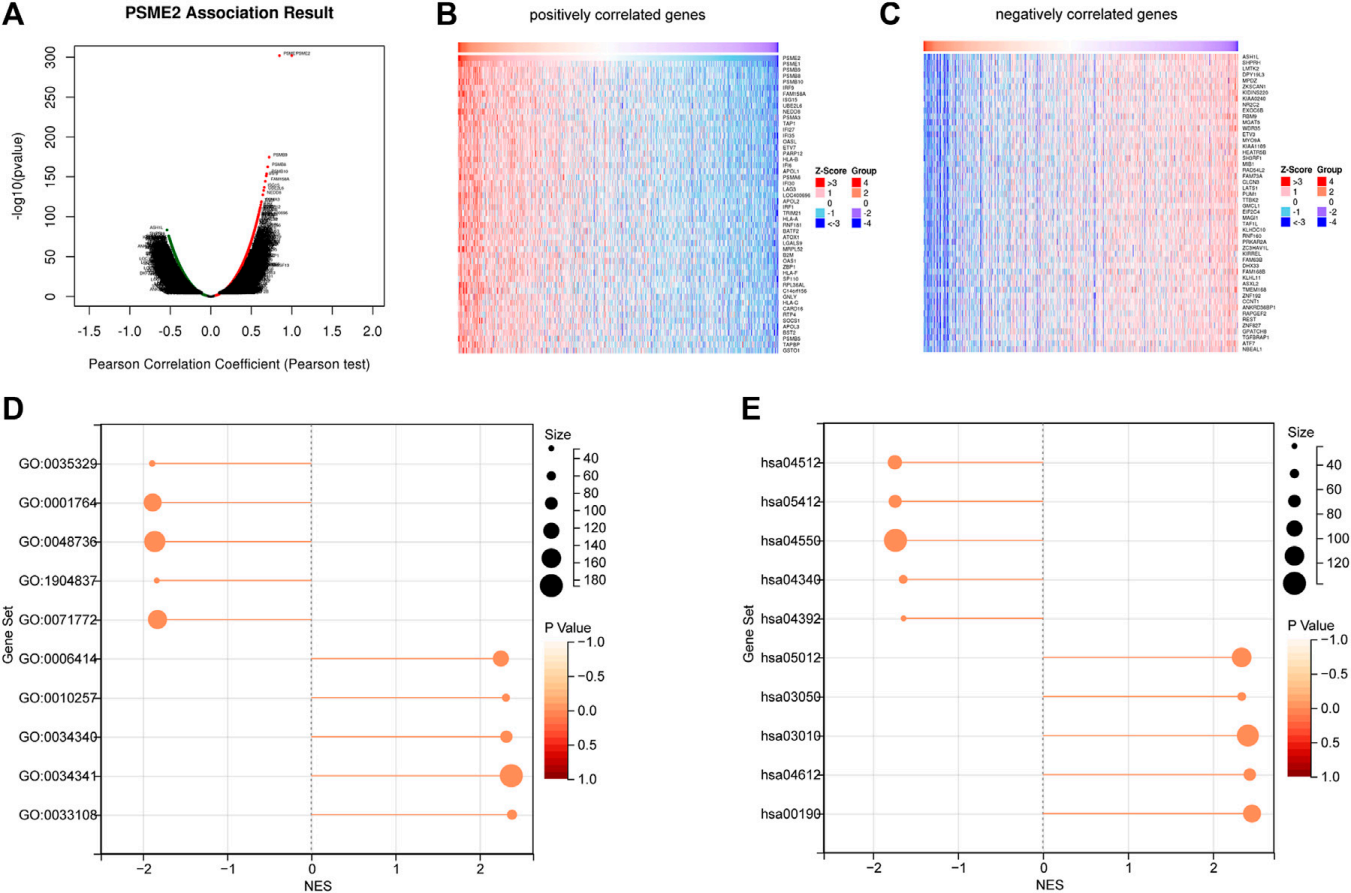
Screening of PSME2-correlated genes and enrichment analysis **(A)** The global PSME2 highly associated genes identified by Pearson test in BrCa **(B)** Heat maps showing top 50 genes positively associated with PSME2 in BrCa **(C)** Heat maps showing top 50 genes negatively associated with PMSE2 in BrCa. Data of A-C was obtained from the TCGA dataset **(D)** BP and **(E)** KEGG enrichment analyses of PSME2-related genes in BrCa.

To investigate the biological features of PSME2 expression in BrCa, we screened the genes that have a correlation with PSME2 expression (Figure 3A). Then, we investigated positively and negatively correlated genes, respectively (Figures 3B, C). To acquire a specific result, PSME2-correlated genes were submitted for BP and KEGG analyses. PSME2 was positively associated with mitochondrial respiratory chain complex assembly, response to interferon-gamma, response to type I interferon, *etc.* in terms of BP enrichment (Figure 3D, Supplementary Table S1), and positively associated with oxidative phosphorylation, antigen processing and presentation, ribosome, *etc.* in terms of KEGG enrichment (Figure 3E, Supplementary Table S2). In general, PSME2 plays a role in many biological processes and is significantly related to immune-related processes in BrCa.

### PSME2 was associated with immuno-hot TIME in BrCa

**FIGURE 4:**
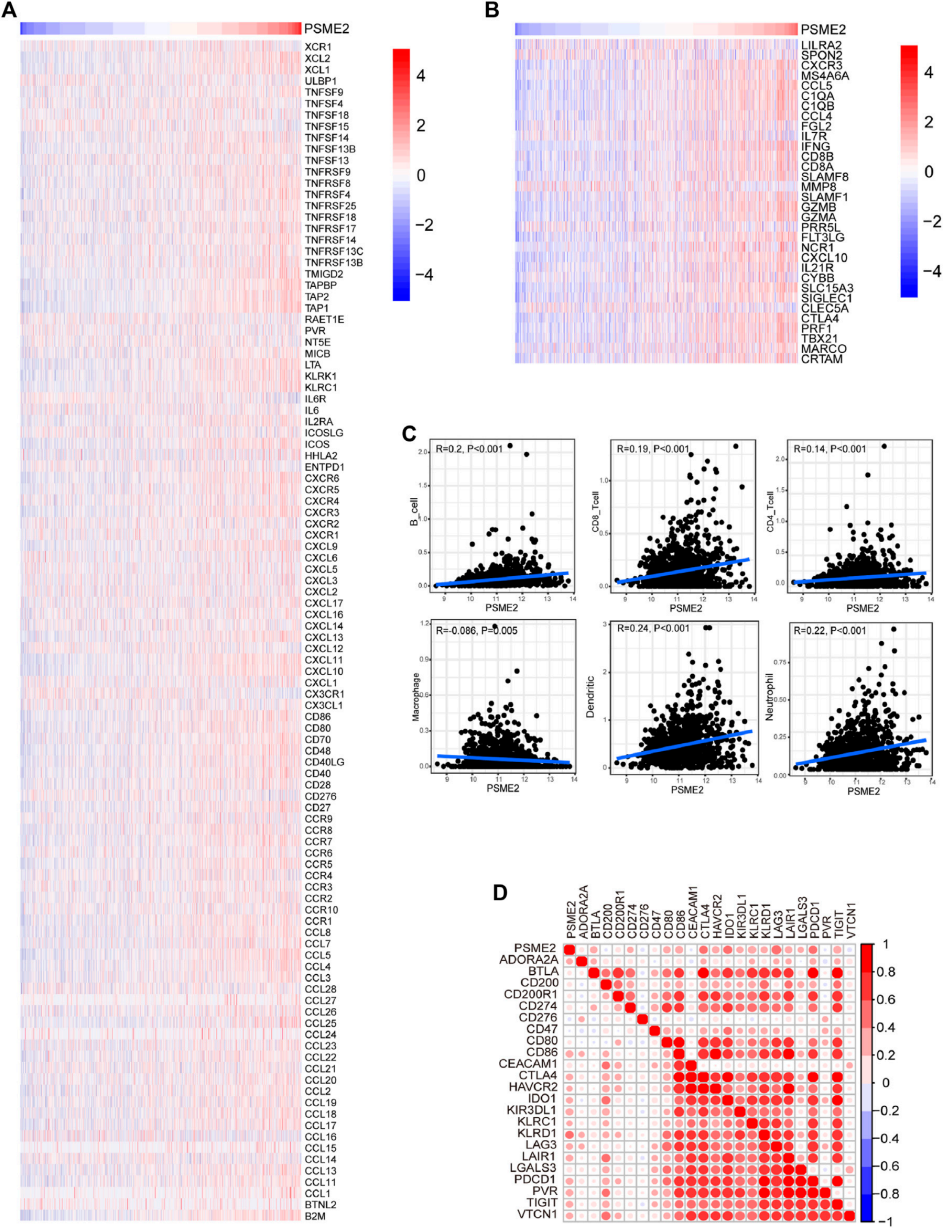
Associations between PSME2 and TIME features in the TCGA dataset **(A)** Expression levels of immunomodulators (chemokines, immunostimulators, MHC, and receptors) in the high- and low-PSME2 groups in BrCa. Red: high expression, blue: low expression **(B)** Expression levels of the gene markers of the common TIICs in the high- and low-PSME2 groups. Red: high expression, blue: low expression **(C)** Correlation between PSME2 and levels of TIICs calculated using the TIMER algorithms **(D)** Correlations between PSME2 and common inhibitory immune checkpoints. The color and the values indicate the Pearson correlation coefficient. Data of Figure 4 was obtained from the TCGA dataset.

Immuno-hot tumors are featured by high levels of TIICs and molecular features of immuno-activation. We performed a series of analyses to investigate the immuno-correlations of PSME2 in BrCa using the TCGA cohort. The result exhibited that PSME2 is positively related to a majority of immunomodulators (including chemokine, MHC, immunostimulator, and receptor) in BrCa (Figure 4A). We also evaluated the gene markers of immune cells and found that these markers were overexpressed in the high PSME2 group (Figure 4B). The expressive abundance of common immune cells is also positively related to the expression of PSME2 expression (Figure 4C). Moreover, PSME2 was also positively correlated with common inhibitory immune checkpoints, such as PD-L1 (Figure 4D). Overall, PSME2 is tightly correlated with the features of immuno-hot TIME in BrCa.

### PSME2 was positively correlated with biomarkers for immunotherapy in BrCa

**FIGURE 5:**
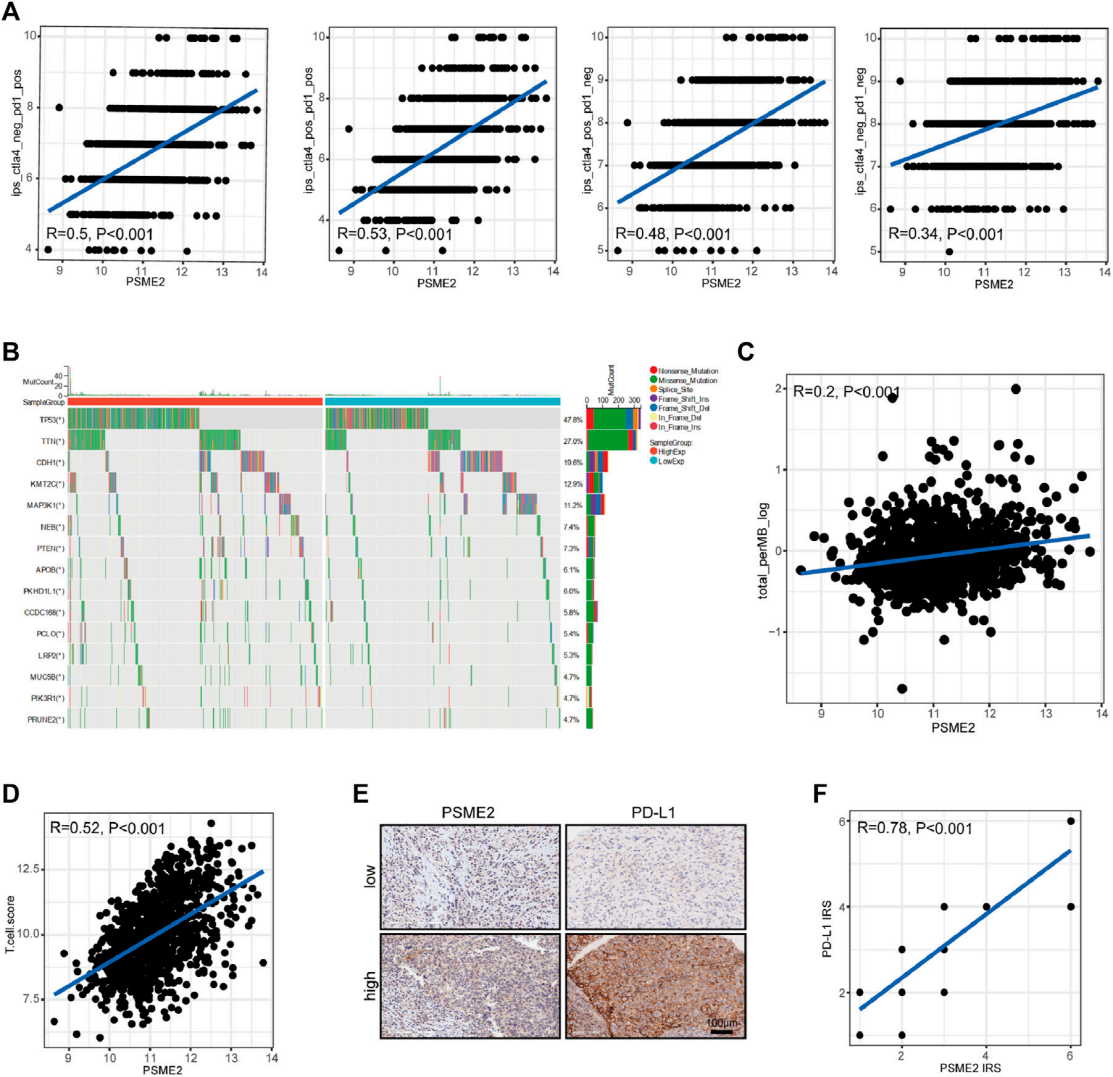
Associations between PSME2 and immunotherapy biomarkers in BrCa **(A)** Positive correlations between PSME2 and the IPS **(B)** The altered landscapes of common genes in BrCa **(C)** Positive correlation between PSME2 and the TMB level **(D)** Positive correlation between PSME2 and the T cell infamed score. Data of A-D was obtained from the TCGA dataset **(E)** Representative images revealing low and high PSME2 and PD-L1 expression using anti-PSME2 staining. Magnification, ×200 **(F)** Positive correlation between PSME2 and PD-L1 protein expression.

Next, we assessed the associations between PSME2 and several biomarkers for immunotherapy, including IPS, TMB, and T cell inflamed score. First, PSME2 expression was positively correlated with IPS (Figure 5A). In addition, genetic alterations were various between the low and high PSME2 groups, which were more frequent in the high PSME2 group (Figure 5B). As expected, PSME2 expression was positively correlated with TMB level in BrCa (Figure 5C). Moreover, the positive correlation between PSME2 and T cell inflamed score was also observed (Figure 5D). To further validate the correlation between PSME2 and TIME features, we conducted IHC staining to visualize PSME2 and PD-L1 expression in BrCa tissues (Figure 5E), and semi-quantitative analysis revealed that PSME2 was positively correlated with PD-L1 expression in BrCa (Figure 5F). To sum up, PSME2 is positively correlated with reported immunotherapeutic biomarkers in BrCa, indicating PSME2 may predict the immunotherapeutic response.

### PSME2 predicted immunotherapeutic response in BrCa

**FIGURE 6:**
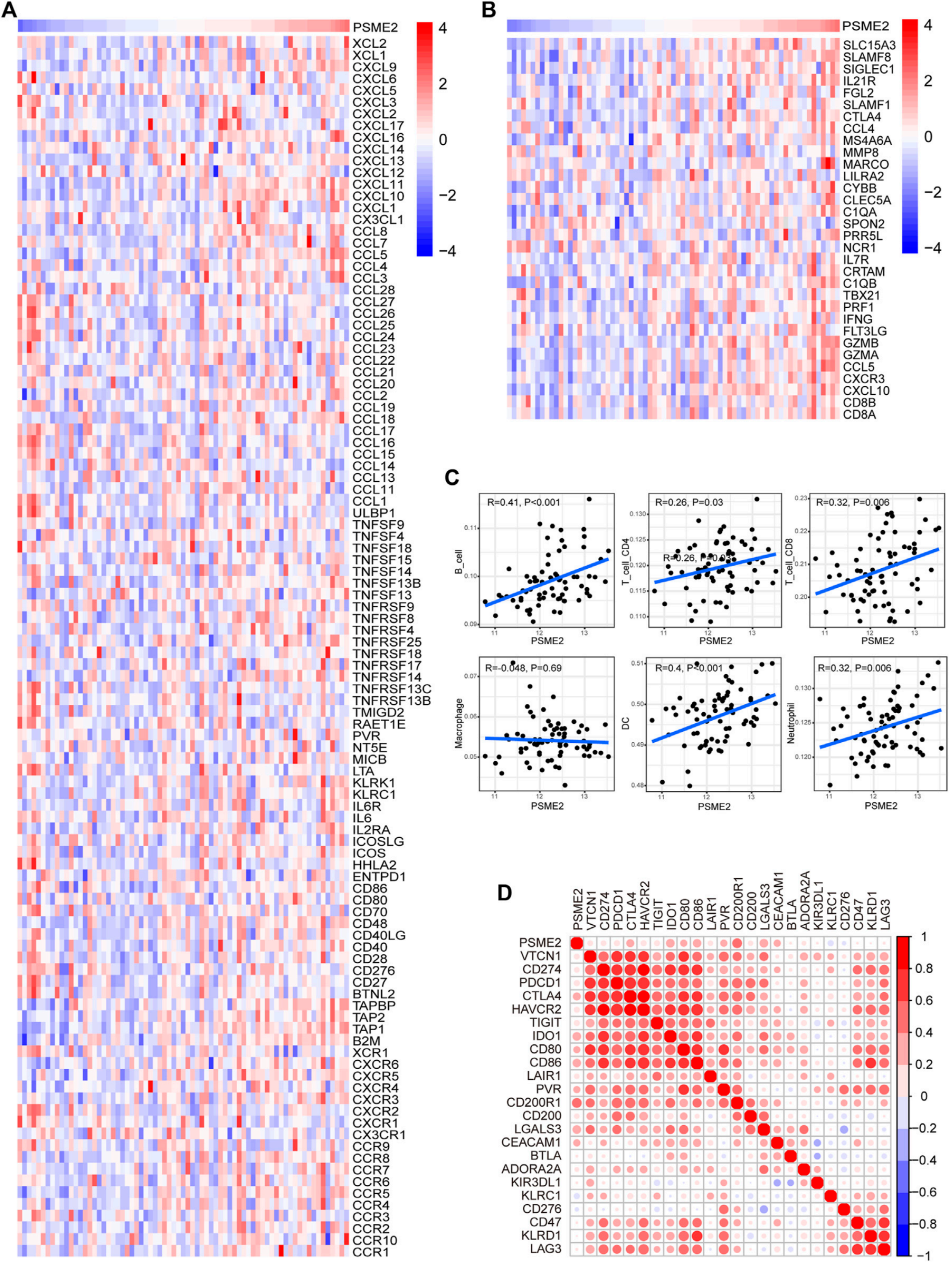
Associations between PSME2 and TIME features in the GSE173839 dataset **(A)** Expression levels of immunomodulators (chemokines, immunostimulators, MHC, and receptors) in the high- and low-PSME2 groups in BrCa. Red: high expression, blue: low expression **(B)** Expression levels of the gene markers of the common TIICs in the high- and low-PSME2 groups. Red: high expression, blue: low expression **(C)** Correlation between PSME2 and levels of TIICs calculated using the TIMER algorithms **(D)** Correlations between PSME2 and common inhibitory immune checkpoints. The color and the values indicate the Pearson correlation coefficient. Data of Figure 6 was obtained from the GSE173839 dataset.

**FIGURE 7:**
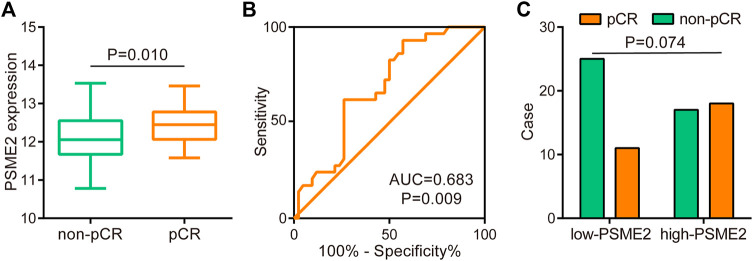
Predictive role of PSME2 in immunotherapy in the GSE173839 dataset **(A)** Expression level of PSME2 in BrCa with various immunotherapeutic responses **(B)** ROC analysis of predictive values of PSME2 in the BrCa immunotherapy cohort. Data of Figure 7 was obtained from the GSE173839 dataset **(C)** Immunotherapeutic response rates between low- and high- PSME2 expression.

Since PSME2 was positively correlated with several reported immunotherapeutic biomarkers, we next explored whether PSME2 could be a novel biomarker for immunotherapy in BrCa using the GSE173839 dataset. Similar to findings of the TCGA dataset, PSME2 was correlated with a majority of immunomodulators, gene markers of immune cells, infiltrating levels of immune cells, and expression levels of inhibitory immune checkpoints (Figures 6A–D). In addition, PSME2 was upregulated in BrCa tissues with pathological complete response (pCR) (Figure 7A), and the predictive value of PSME2 was satisfactory (Figure 7B). In addition, BrCa patients with high PSME2 expression showed higher response rate in the GSE173839 cohort (Figure 7C). Overall, PSEM2 is related to the features of immuno-hot TIME and could predict the immunotherapeutic response in BrCa.

## Discussion

According to previous studies, PSME2 plays the role of promoting tumor progression in clear cell renal cell carcinoma, which is positively correlated with the ability of invasion regulating BNIP3-mediated autophagy (Wang et al., 2021). In addition, increasing numbers of studies showed that PSME2 may be used as a tumor biomarker. Compared with paratumor tissues, the expression of PSME2 is upregulated in tumor tissues in multiple cancer types, such as gastric cancer (Guo et al., 2021), skin cutaneous melanoma (Wang et al., 2019), and urothelial cancer (Wang et al., 2020). In the current research, we utilized the TCGA dataset to analyze the expression and prognostic values of PSME2 in BrCa, we found that PSME2 was overexpressed in BrCa tissues but positively related to better prognosis.

Given the discrepancy between expression and prognostic value, we next explored underlying mechanisms in BrCa. As result, PSME2 was positively related to immune-related processes. More importantly, PSME2 expression was positively correlated with immunomodulator and infiltrating immune cells in tumors, which are main components of TIME (Halbert and Einstein, 2021). All results indicated that PSME2 played a significant role in the immuno-hot TIME. For further research, we assessed the relationship between PSME2 and biomarkers for immunotherapy. We found PSME2 exhibits a positive correlation with biomarkers of immunotherapy.

Recently, increasing evidence has proved that TIME determines the responses to multiple anti-tumor therapies, especially immunotherapy (Bonaventura et al., 2019). immunomodulators and TIICs are main components of the TIME, which are heterogeneous and dynamic (Huang et al., 2021). On account of the features of the TIME, tumors could be divided into two subtypes, which included immuno-hot and immuno-cold tumors. Immuno-cold tumors are featured by immuno-suppressive TIME and the lack of TIICs infiltration, and most solid immune-cold tumors are not responsive to immunotherapy. Contrarily, immuno-hot tumors are potential candidates which are responsive to immunotherapy (Qin et al., 2019b). Thus, understanding the constitution of TIME within which immune cells function and identification of potential biomarkers related to the features of the TIME is significant for the discrimination of beneficiaries from immunotherapy in clinical practice (Chen et al., 2021).

Previous studies also reported the immuno-correlated role of PSME2 in other cancers. Guo *et al.* reported overexpressed PSME2 was notably associated with better prognosis, the infiltration levels of common immune cells, and TMB level (Guo et al., 2021). In addition, PSME2 and other four gene markers for malignant cells (ARID5A, SERPINE2, GPC3, and S100A11) were combined to develop a prognostic signature in melanoma (Kang et al., 2022). More importantly, PSME2 was a novel tumor-associated antigen, which was overexpressed, amplified, and mutant in BrCa (Li et al., 2022) and contributed to a novel prognostic signature (Dong et al., 2022). Overall, PSME2 was tightly immuno-correlated in not only BrCa, but also multiple cancer types, which may be a pan-cancer biomarker.

Up to date, a large number of studies have uncovered multiple genes can serve as biomarkers for immunotherapy response in BrCa or other cancer types, such as CCDC69 (Yi et al., 2022), TIMM8A (Zhang et al., 2022b), and ACE2 (Mei et al., 2022). Most of these studies are lacking real-world cohort validation, which is also the main shortcoming of our current research. However, the defect of PD-L1 as a biomarker could not be overlooked as well. Previous studies have revealed that PD-L1 is heavily glycosylated and deglycosylation significantly enhances PD-L1 positive rate using IHC examination (Lee et al., 2019; Mei et al., 2021). Overall, given the defect of PD-L1 as a biomarker, PSME2, along with other biomarkers may be used as significant complementary biomarkers.

## Conclusion

In this report, we comprehensively analyzed the expression and immuno-correlations of PSME2 in BrCa and found that PSME2 was associated with well prognosis and immuno-hot TIME in BrCa. Moreover, PSME2 was positively correlated with biomarkers for immunotherapy and even could predict immunotherapeutic response in BrCa. Overall, we reported PSME2 as a novel indicator for prognostic assessment and superior population discrimination for immunotherapy in BrCa.

## Data Availability

The original contributions presented in the study are included in the article/Supplementary Material, further inquiries can be directed to the corresponding author.
