# Supplementary material for: PSME2 identifies immune-hot tumors in breast cancer and associates with well therapeutic response to immunotherapy
Source: Front Genet. 2022 Dec 13;13:1071270. doi: 10.3389/fgene.2022.1071270 (PMC9793949; doi:10.3389/fgene.2022.1071270)
Supplement: Supplementary file 1 [file Table1.DOCX]

**Table S1. BP enrichment analysis of PSME2-related genes in BrCa (Related to Figure 3D).**

| Gene set | Description | NES | P value |
| --- | --- | --- | --- |
| GO:0033108 | mitochondrial respiratory chain complex assembly | 2.385 | <0.001 |
| GO:0034341 | response to interferon-gamma | 2.377 | <0.001 |
| GO:0034340 | response to type I interferon | 2.318 | <0.001 |
| GO:0010257 | NADH dehydrogenase complex assembly | 2.313 | <0.001 |
| GO:0006414 | translational elongation | 2.253 | <0.001 |
| GO:0035329 | hippo signaling | -1.890 | <0.001 |
| GO:0001764 | neuron migration | -1.884 | <0.001 |
| GO:0048736 | appendage development | -1.859 | <0.001 |
| GO:1904837 | beta-catenin-TCF complex assembly | -1.835 | <0.001 |
| GO:0071772 | response to BMP | -1.827 | <0.001 |
