# Supplementary material for: PSME2 identifies immune-hot tumors in breast cancer and associates with well therapeutic response to immunotherapy
Source: Front Genet. 2022 Dec 13;13:1071270. doi: 10.3389/fgene.2022.1071270 (PMC9793949; doi:10.3389/fgene.2022.1071270)
Supplement: Supplementary file 2 [file Table2.DOCX]

**Table S2. KEGG enrichment analysis of PSME2-related genes in BrCa (Related to Figure 3E).**

| **Gene set** | **Description** | **NES** | **P value** |
| --- | --- | --- | --- |
| hsa00190 | Oxidative phosphorylation | 2.460 | <0.001 |
| hsa04612 | Antigen processing and presentation | 2.434 | <0.001 |
| hsa03010 | Ribosome | 2.411 | <0.001 |
| hsa03050 | Proteasome | 2.340 | <0.001 |
| hsa05012 | Parkinson disease | 2.338 | <0.001 |
| hsa04512 | ECM-receptor interaction | -1.742 | <0.001 |
| hsa05412 | Arrhythmogenic right ventricular cardiomyopathy | -1.739 | <0.001 |
| hsa04550 | Signaling pathways regulating pluripotency of stem cells | -1.735 | <0.001 |
| hsa04340 | Hedgehog signaling pathway | -1.644 | 0.009 |
| hsa04392 | Hippo signaling pathway | -1.639 | <0.001 |
